# ‘A Wonderfull Monster Borne in Germany’: Hairy Girls in Medieval and Early Modern German Book, Court and Performance Culture*

**DOI:** 10.1111/glal.12054

**Published:** 2014-09-24

**Authors:** MA Katritzky

**Affiliations:** The Open University

## Abstract

Human hirsuteness, or pathological hair growth, can be symptomatic of various conditions, including genetic mutation or inheritance, and some cancers and hormonal disturbances. Modern investigations into hirsuteness were initiated by nineteenth-century German physicians. Most early modern European cases of hypertrichosis (genetically determined all-over body and facial hair) involve German-speaking parentage or patronage, and are documented in German print culture. Through the Wild Man tradition, modern historians routinely link early modern reception of historical hypertrichosis cases to issues of ethnicity without, however, recognising early modern awareness of links between temporary hirsuteness and the pathological nexus of starvation and anorexia. Here, four cases of hirsute females are reconsidered with reference to this medical perspective, and to texts and images uncovered by my current research at the Herzog August Library and German archives. One concerns an Italian girl taken to Prague in 1355 by the Holy Roman Empress, Anna von Schweidnitz. Another focuses on Madeleine and Antonietta Gonzalez, daughters of the ‘Wild Man’ of Tenerife, documented at German courts in the 1580s. The third and fourth cases consider the medieval bearded Sankt Kümmernis (also known as St Wilgefortis or St Uncumber), and the seventeenth-century Bavarian fairground performer Barbara Urslerin.

Krankhafter menschlicher Hirsutismus kann aufgrund unterschiedlicher Ursachen auftreten, zu denen u.a. genetische Veränderungen und Vererbung, verschiedene Krebserkrankungen und hormonelle Störungen gehören. Die moderne Hirsutismus-Forschung ist im 19. Jh. von deutschen Forschern initiiert worden. Die meisten europäischen frühneuzeitlichen Erscheinungen von Hypertrichose (dem genetisch bedingten Haarwuchs am gesamten Körper und im Gesicht) gehen auf deutschsprachige Eltern oder Förderer zurück und sind in Deutschland in den Druck gelangt. Bei Untersuchungen des Motivs des Wilden Mannes zieht die aktuelle geschichtswissenschaftliche Forschung in der Regel Verbindungslinien zwischen der frühneuzeitlichen Wahrnehmung von Hypertrichose-Fällen und Fragen der Ethnizität, ohne jedoch zu beachten, dass in der Frühen Neuzeit die Verbindung zwischen temporärem Hirsutismus und der krankhaften Verknüpfung von Unterernährung und Anorexie bekannt war. Im vorliegenden Beitrag werden vier Fälle von an Hirsutismus erkrankten Frauen neu analysiert, unter Einbezug dieser medizinischen Perspektive und unter Beachtung von Texten und Abbildungen, die meine jüngsten Forschungen in der Herzog August Bibliothek und an deutschen Archiven ans Licht gefördert haben. Die hier betrachteten Fälle betreffen ein italienisches Mädchen, das 1355 von Anna von Schweidnitz, Kaiserin des Hl. Römischen Reichs, nach Prag gebracht wurde; Madeleine und Antonietta Gonzalez, die Töchter des ‘Wilden Manns’ von Teneriffa, die in den 1580er Jahren an deutschen Höfen bezeugt sind; die bärtige Sankt Kümmernis (Wilgefortis), und die bayerische Jahrmarktkünstlerin Barbara Urslerin.

Through the Wild Man tradition, modern historians routinely link early modern reception of historical hypertrichosis cases to issues of ethnicity without, however, recognising early modern awareness of links between temporary hirsuteness and the pathological nexus of starvation and anorexia. Here, four cases of hirsute females are reconsidered with reference to this medical perspective. Hildegard von Bingen (1098–1179) notes of a group of sinners featured in one of her religious visions: ‘Quidam vero in corpore hirsuti et in anima multa immunditia humanæ pollationis perfusi sordebant.’^1^ Her description affords powerful insights into medieval receptions of pathological hirsuteness and its most extreme human manifestation, hypertrichosis, a rare genetic condition whose symptoms typically include serious dental defects as well as all-over body and facial hair. Even by the time of *Twelfth Night*, Shakespeare’s questioning of external appearance as a barometer of moral behaviour was still exceptional:

In Nature, there’s no blemish but the minde:

None can be call’d deform’d, but the vnkinde.

Vertue is beauty, but the beauteous euill

Are empty trunkes, ore-flourish’d by the deuill.^2^

Pathological hirsuteness, integral to several of the classical anthropological categories often referred to as the marvellous races, is reported by many early modern travellers, either in connection with whole populations, or, as in a travel account of 1590 depicting a fearsomely bearded man covered with thick fur from the top of his head to his elbows and upper thighs, for individual cases:

In the court of Prester John, there is a wilde man, and another in the high streete at Constantinople […] and all ouer their bodies they haue wonderfull long haire, they are chained fast by the necke, and will speedely deuour any man that commeth in their reach.^3^

Despite the rarity of pathologically hirsute humans, the considerable and diverse presence of what may be called hairy folk in early modern culture is especially marked for hypertrichosis. Building on a range of existing critical methodologies, I am developing a new approach to researching complex pre-modern receptions and representations of monstrous, disabled, and diverse corporeality such as those reflected by St Hildegard or Edward Webbe. My term ‘literary anthropologies’ refers to physically diverse human and near human categories based on literature, folklore or mythology. When trying to make sense of early modern perceptions of hairy humans and near humans, it seems that how early modern people, including medical and theatre practitioners, thought about the diverse cultural representations of excessively hairy folk such as classical satyrs, werewolves, Early Christian anchorites or medieval Wild Men, is key to evaluating how they saw, depicted and described fellow citizens with hypertrichosis and related pathological conditions.

Here, I focus on four cases of pathologically hairy girls born in or with strong links to Germany, and suggest that we can enhance our understanding of them by considering them in the context of ‘literary anthropologies’. My second, third and fourth case studies consider the daughters of Pedro Gonzalez and two bearded women, the medieval Sankt Kümmernis and seventeenth-century Barbara Urslerin, with reference to my literary anthropologies approach and new ways of thinking about early modern receptions of hypertrichosis and the medieval Wild Man tradition. Significantly, my first example represents a fourteenth-century precedent for German court patronage of non-German-born hypertrichosis cases.

## 1. The Tuscan girl of 1355

In 1353, fourteen year-old Anna von Schweidnitz (1339–62) became the third of the four wives of Emperor Charles IV (1316–78). The following year, the imperial couple travelled from Prague to Rome, and in April 1355, Charles was crowned Holy Roman Emperor in Rome. During their return journey, according to the Florentine chronicler Mattea Villani:

Mentre che l’imperadore era a Pietrasanta, per grande maraviglia, e cosa nuova e strana, gli fu presentata una fanciulla femmina d’età di sette anni, tutta lanuta come una pecora, di lana rossa mal tinta, ed era piena per tutta la persona di quella lana insino all’ estremità delle labra e degli occhi. L’imperadrice, maravigliatasi di vedere un corpo umano così maravigliosamente vestito dalla natura, l’accomandò a sue damigelle che la nudrissono e guardassono, e menolla nella Magna.^4^

That sixteen year-old Empress Anna was indeed sufficiently fascinated by this woolly-haired seven year-old Tuscan girl to take her back to Prague is confirmed by the Bishop of Bisignano, Giovanni de’Marignolli (1290–1357). Engaged in 1355 by Emperor Charles IV to work on the *Annals of Bohemia*, this former delegate to the Emperor of China from Pope Benedict XI’s papal court at Avignon, interpolated his contributions to the imperial chronicle with Far Eastern reminiscences. His anthropologically astute note confirming that the Tuscan girl was both a redhead and a unique case of hypertrichosis in her own community is closely followed by a passage identified as the earliest European eye-witness account of the indigenous Veddar tribe of Sri Lanka,^5^ where Marignolli was marooned during 1349:

So the most noble Emperor Charles IV brought from Tuscany a girl whose face, as well as her whole body, was covered with hair, so that she looked like the daughter of a fox! Yet is there no such race of hairy folk in Tuscany: nor was her own mother even, nor her mother’s other children so, but like the rest of us. […] We do not suppose that such creatures exist as a species, but regard them as natural monstrosities. […] The truth is that no such people *do* exist as nations, though there may be an individual monster here and there. […] There are also wild men, naked and hairy, who have wives and children, but abide in the woods. They do not show themselves among men, and I was seldom able to catch sight of one; for they hide themselves in the forest when they perceive anyone coming.^6^

The Tuscan girl attracted Europe-wide attention. She is recorded in Montaigne’s *Essayes*: ‘There was also presented vnto *Charles* king of *Bohemia*, an Emperour, a young girle, borne about *Pisa*, all shagd and hairy over and over.’^7^ In 1570, Antonio de Torquemada implies strong connections between hypertrichosis and diabolical generation, linking her to a boy shown around sixteenth-century Spain for money by his father:

I haue heard of a woman deliuered of a child all couered ouer with rough haire, the reason wherof was, that she had in her chamber the picture of Saint Iohn Baptist clothed in hairy skinns, on which the woman vsing with deuotion to contemplate, her chyld was borne both in roughnes & figure like vnto the same. […] Marcus Damascenus writeth […] that it hapned […] neere the Citty of Pysa [*marginalium*: ‘the place is called *Petra sancta*’] It is not long since that there went through Spayne a man gathering money, with the sight of a son of his couered with hayre, in such quantity so long & thicke, that in his whole face there was nothing els to be seen but his mouth and eyes: Withall, the haire was so curled, that it crimpled round like Ringes, and truly the wilde Sauages which they paynt, were nothing so deformed, and ouer their whole body so hairie as was thys boy.^8^

Torquemada directly follows his account of these two examples of hirsuteness with a detailed report of ‘A wonderfull Monster borne in Germany’, fathered by a German actor who refused to remove his devil costume before sleeping with his wife:

I will neyther wonder at this, nor at any such like, seeing that in this our time it is known & affirmed for a matter most true, that certaine Players shewing of a Comedy in Germany, one of them which played the deuill, hauing put on a kinde of attyre most grisly and feareful, whe[n] the Play was ended went home to his own house, where taking a toy in the head, he would needs vfe the company of his wife without changing the deformed habite hee had on, who hauing her imagination fearefully fixed on the ouglie shape of that attire with which her husband was the[n] clothed, conceaued childe, and came to be deliuered of a creature representing the very likenes of the deuill, in forme so horrible, that no deuil of hell could bee figured more lothsome or abhominable.^9^

A similar monstrous baby was said to have been fathered directly after a religious procession in s’Hertogenbosch:

*Margaret* Daughter to the Emperour *Maximilian* the first, told the Ambassadour of *Ferdinand* King of *Hungary*; that at *Tsertoghenbosch* a City in *Brabant*, in a procession upon a solemn Festival; some of the Citizens went disguised according to the custom of the place: (some in the habit of Angels, and others in the shape of Devils as they are painted) one of these Devils having play’d his gambols a great while; ran home to his House in his Devils attire, took his Wife, threw her upon a bed, saying that he would get a young Devil upon her. He was not much deceiv’d, for of that copulation, there was born a child, such as the wicked Spirit is painted.^10^

Limiting herself to French cases, Jody Enders insightfully discusses variants of this ‘urban legend’, notably one featuring a Devil actor in the French town of Bar-le-Duc, whose wife bore him a baby in 1485 whose upper half resembled a devil, in the context of the then widely perceived ‘monstrosity that lies at the heart of theater’.^11^ Such diabolical connotations meant that accidents connected with Wild Man and devil costumes were widely interpreted as divine punishment. The most notorious such event, still considered newsworthy in Germany in 1707,^12^ occurred in January 1392, when four noble carnival masqueraders costumed as Wild Men at the Bal des Ardents accidentally burned to death, and King Charles VI of France, who had participated in a similar ball in 1389, was seriously injured.^13^ Similar German conflagrations include one at Waldenburg Castle in Württemberg, on 7 February 1570.^14^ The noble carnival masqueraders who died of their injuries when their devil costumes caught fire included the host, Duke Eberhard von Hohenlohe-Waldenburg (1635–70) and his brother-in-law Georg III von Tübingen. Others, including Valentin von Berlichingen, Simon von Neudeck and Duke Albert von Hohenlohe survived burns whose extreme severity had led highly-qualified medical consultants to diagnose them as the incurable result of poisonous hellfire. At the court of Georg von Schleinitz’s bride, at Wickenthal near Meissen in Saxony, he and five other noblemen (and his bride, in trying to extinguish the flames) burned to death when their shaggy bear costumes caught fire during his wedding festivities.^15^

The Tuscan girl’s hypertrichosis was attributed to non-diabolical maternal influence, as reported by Torquemada and other sixteenth-century commentators, such as the French court surgeon Ambroise Paré:

*Damascene* reports that he saw a maide hairy like a Beare, which had that deformity by no other cause or occasion than that her mother earnestly beheld, in the very instant of receiving and conceiving the seed, the image of St. *John* covered with a camells skinne, hanging upon the poasts of the bed.^16^

Paré’s outrageously inauthentic woodcut depiction of this Tuscan girl was widely copied. Similar portraits of her were published by Ulysses Aldrovandi in 1642,^17^ and in numerous editions of *Aristotle’s masterpiece*, often with an illustration of a short-lived late-sixteenth-century hirsute boy.^18^ As the most widely read pre-modern English medical guide, continuously in print from 1684 to the 1930s, *Aristotle’s masterpiece* ensured that Paré’s fanciful metamorphosis of the woolly-haired seven year-old Tuscan girl into a hirsute, nude, fully mature woman became the most iconographically influential of all book illustrations of hypertrichosis cases.

## 2. Madeleine and Antonietta Gonzalez

Visual and textual records of the daughters of Pedro Gonzalez are far more diverse. Modern historical considerations of pathologically excessive human hairiness were inaugurated in the 1870s, by Max Bartels’s rambling, monograph-length, three-part article. Peppered with case studies from his own Berlin practice, his chronological tables document hypertrichosis cases dating from the sixteenth to the nineteenth centuries.^19^ Although Bartels’s text does briefly allude to the Tuscan girl, his tables omit this and other early cases widely reported by sixteenth-century authorities, such as the report of Albertus Magnus, in *De animalibus*, of a Wild Couple captured in thirteenth-century Saxony, or the hirsute boy said to have been born in Rome in 1282 to a papal concubine who had looked at pictures of bears during her pregnancy.^20^ Following Bartels’s lead, medical historians still widely regard the sixteenth-century Gonzalez family as the earliest example of historically-documented hypertrichosis.

Pedro Gonzalez was born in the 1530s on Tenerife in the Spanish Canary Islands, and taken as a child to the French court. His marriage to Catherine, a young Frenchwoman with normal hair, produced at least seven children. The couple’s eldest and youngest sons Paolo (born 1570s) and Ercole, born in Parma in 1595, and daughter Francesca (born *c*.1582) were non-hirsute. However, Pedro’s hypertrichosis was inherited by Madeleine (born *c*.1575), Henri (*c*.1580–1656), Antonietta (born *c*.1588) and Orazio, born in Parma in 1592, and persisted at least into the third generation.^21^ Their physical symptoms closely corresponded to perceptions of the medieval Wild Man tradition. The growing family was in fact kept as living ‘Wild Men’ at various courts with Habsburg connections, moving from the French royal court, via Flanders and the German-speaking alpine regions, to the ducal court of Parma, and finally to the Farnese country villa at Capodimonte. Duke Wilhelm V of Bavaria’s attempts to acquire a live Wild Man date back to at least 1571, when Duke Philipp zu Hanau responded to his enquiries with assurances that there were none in his forests.^22^ During the 1580s, the Bavarian and central European Habsburg courts exhibited an extraordinary flurry of interest in the Gonzalez family. The court of Bavaria hosted the Gonzalez family themselves – according to my reading of the evidence – or merely paintings but no actual people according to others. In 1583, Duke Wilhelm wrote to Archduke Karl’s wife, his sister Maria, in Vienna:

Was meine wilde kerle antrifft, will ich die ganz leng abmalen lassen und euchs hineinschickhen. Ich hab auch in Franckhreich geschriben umb all sein herkomen, thun und lassen. Er wirdt aber selbs wenig wissen, dann er gar klein herauskommen und dem könig geschennckht worden. Seindt sonst nit wildt, wie man sy haisse. Der mann ist gar ain feiner beschaidner und höflich[er] gesell, allein das er so zottel ist. So ist das dierndel gar fein und wolzog[en], wann es die haar nit hett im gesicht, wer es ein schöns dierndel. Der pueb kan nit redden, der ist gar narrisch und khurzweilig. Des allten vatter und muetter sein nit rauch, sonder wie ander leüth, und ist mir recht, so sein sy Spanier gewest. Des allten conterfedt will ich auch schickhen, hab die ganz leng, ist nit ain grosse person.^23^

Together with my third case, the seventeenth-century Barbara Urslerin, and their own father Pedro and brother Arrigo, the Gonzalez sisters are depicted in *Der rauch-behaarte Mensch*, a woodcut illustration published in 1685 in a volume of journalistic writings by Eberhard Werner Happel (1647–90).^24^ This composite plate unites seven images based on previous iconographic sources. Its central figure represents an example of the legendary sub-human hairy-bodied bipedal creature known on the Indonesian island of Borneo as the *Ourang Outan*, or ‘wild man of the woods’, while the lowest level features a hairy nude member of the classical ‘marvellous race’ known as the Himantopodes.^25^ Situating portraits of Barbara Urslerin at her harpsichord and four hirsute Gonzalez family members (Pedro, a son and two daughters), within this cultural context, this print makes strong connections between hypertrichosis and hairy-bodied peoples and creatures of classical mythology and fable, the medieval traditions of the hairy anchorite and the Wild Man, and non-Europeans, and raises wider implications for the early modern classification, representation and reception of hypertrichosis sufferers.

Hypertrichosis cases illuminate early modern ideas about definitions and borders of the human – with respect to the supernatural, the zoological natural world, and indigenous non-Europeans. Humanist ethnographical investigations, prompted by the discovery and exploration of Old and New World peoples and species, gave hirsute humans and the Wild Man tradition renewed relevance in early modern medical, cultural and popular circles. The classical satyr drama, or pastoral, was revived not least in order to address such issues. Records such as Happel’s print of 1685 indicate that, despite its numerous non-hirsute members, popular perceptions of the Gonzalez family as ‘Wild Men’, or as a discrete hairy tribe of the type of the classical marvellous races, persisted throughout the seventeenth century. In 1690, for example, the Restoration dramatist Thomas D’Urfey wrote:

*Pliny* and *Solinus* make mention of diverse Hairy Nations and *Lycosthenes* Writes of a certain Island, the Inhabitants whereof have all their Parts, except their Faces and Palms of their hands, cover’d over with long Hair; part of the Hide of such a Savage, a certain Sarmatian sent unto *Ulisses Aldrovandus*, and is kept in the *Musæum* of the Bononian Senate: These kind of Wild Men were first seen at *Bononia*, when the beautiful Marchioness of *Soranium* coming thither, was nobly receiv’d by the *Illustrissimo Marcus Casalius*, who brought with her a Hairy Girl of eight Years of Age, being the Daughter of a Wild Man born in the *Canaries*, whose Effigies [*marginalium: Aldrovand*. in *Monst.Hist*.] *Aldrovandus* expos’d to the view of all his Friends as a great Rarity; there are as *Eusebius* also writes, in the East and West *Indies*, Wild Men who are born smooth like our Infants, but as they grow up have Hair covering their whole Bodies.^26^

Extending, as was then commonplace, the classical anthropological concept of marvellous races to America as well as Asia, D’Urfey here refers to Antonietta Gonzalez in the context of Isabella Pallavicina, Marchesa di Soragna, the noble patron who adopted her at the age of eight. Detailed coloured drawings of Antonietta and her relatives, commissioned by the Bolognese physician Ulysses Aldrovandi, formed the basis for the woodcuts in his posthumous treatise of monstrous and pathologically abnormal humans and animals of 1642, and later prints, such as Happel’s contextualisation of them with Wild Men.^27^

## 3. Sankt Kümmernis

Wild Men were consistently characterised in medieval and early modern art and literature by their shaggy-haired body, inarticulate lack of human speech and liminal humanity.^28^ My attempts to get beyond modern interpretations of the Wild Man as ‘a purely mythic creature […] a literary and artistic invention of the medieval imagination’,^29^ are greatly aided by a close reading of a posthumously published Lenten sermon of Johann Geiler von Kaysersberg (1445–1510). The renowned Swiss preacher subdivides Wild Men into five categories that illuminate early modern perceptions of hirsuteness: pygmies, anchorites, devils, satyrs and Spaniards.^30^ In the context of scholarly enquiries into hairy folk and Wild Men, earnest attempts have been made to get to grips with their apparent disparate randomness. Here, I focus on hirsuteness.

Initially, where genetically inherited, hypertrichosis is caused by a specific dominant gene mutation, passed down from one generation to the next in much the same way that brown eyes are dominant over blue eyes (except, of course, that given its extreme rarity, no human child will ever have more than one parent with hypertrichosis). Because everyone who inherits the gene exhibits the physical symptoms, only parents with visible hypertrichosis can pass it on to their children, and their children have only a fifty percent chance of inheriting, manifesting, and being able to pass on, the condition. Previously overlooked in this context is that hypertrichosis is by no means exclusively a life-long symptom of one specific genetic condition. HTLA (*hypertrichosis lanuginosa acquisita*), a form of non-congenital hypertrichosis sometimes confined to limited areas of the body, is indicative of certain cancers, and can be symptomatic of a quite unrelated group of conditions: the nexus of severe malnutrition, starvation and anorexia nervosa. Mainly through extreme poverty or piety, anorexia or starvation-related temporary hypertrichosis was widely recognised in the medieval and early modern periods, and, mainly due to gender-related body image issues, it is manifesting regularly again in modern Europe.^31^ Temporary pathological hirsuteness, diagnosed as a symptom of malnourishment and anorexia by modern physicians^32^ and noted in this medical context by historians of modern disasters such as the nineteenth-century Irish Potato Famine, has not previously been considered in relation to the medieval Wild Man tradition.

One of Geiler von Kaysersberg’s five categories of Wild Man, the hermit (‘solitari’), acknowledges the tradition’s debt towards ‘the legend of the hairy anchorite’, which also informs the Himantapodes depicted in Happel’s print of 1685. Concerning certain holy Christians whose bodies were said to have been covered all over by long hair, this legend is rooted in accounts of hairy-bodied desert-inhabiting Semitic demons, and Old Testament Hebrew characters such as Samson, Nebuchadnezzar, Ishmael and Esau. For some Christians, such as St Onofrius or St Paul of Thebes, the condition was said to have become a permanent physical manifestation of divine grace. Others, such as St James, St John Chrysostom or Mary Magdalene, were said to have grown furry hair all over their body as a temporary penance for sinfulness. In typical Italian fashion, Donatello shows Mary Magdalene clothed only with her long, thick tresses; however some German artists, such as Tilman Riemenschneider,^33^ depicted the penitent saint with her nude body covered with soft fur in the manner of a Wild Woman, leaving only her face and neck, breasts, knees and hands bare.

HTLA-related symptoms, hardly noted in pre-modern contexts, plausibly explain the amenorrhoea and masculinisation of Hipppocrates’s previously fertile Phaethousa, who ‘grew hairy all over, she grew a beard’ and shortly thereafter died, while grieving for her exiled husband Pytheas;^34^ or the temporary hirsuteness of literary heroines such as Raue Else, heroine of the medieval epic ‘Wolfdietrich’, a furry Wild Woman who approaches the hero on all fours but recovers her former smooth skin and civilised identity as Princess Sigeminne on visiting a Fountain of Youth.^35^ HTLA also explains the pathological hirsuteness of malnourished female early Christian fasters such as Mary Magdalene, Mary of Egypt, and medieval saints who became bearded in response to aggressive attacks on their chastity: the Italian St Galla, Spanish St Paula of Avila, and Portuguese septuplet St Wilgefortis. According to some Tirolean accounts of Wilgefortis, known as St Uncumber in England, and, despite her non-canonical status, widely revered as Sankt Kümmernis in Bohemia, Bavaria and Alpine Germany, this bearded saint also grew all-over body hair.^36^ In 1963, two physicians made the connection between Wilgefortis’s unusual repulsion of her unwanted suitor, the King of Sicily, and a modern medical case history published by endocrinologists in 1959, of a twenty-three year-old woman who: ‘confronted with the stress of an impossible extra-marital situation, developed, within the space of little more than a month, a luxurious growth of body hair’.^37^ While the 1959 publication refers neither to historically nor hagiographically documented cases, what it labels ‘idiopathic hirsutism’ is also cited as a possible explanation for the case of St Wilgefortis by Schulenberg.^38^ Exceptionally, the physician J. Hubert Lacey’s consideration of Wilgefortis explicitly connects her beard and secondary growth of lanugo (pre-natal) hair to medical symptoms of fasting and anorexia.^39^ Rudolph Bell discusses anorexia with reference to ascetic female saints, 40 but neither he nor Lacey consider the hairy anchorite or Wild Man traditions, and Lacey’s central insight remains unacknowledged in later studies of pathologically hirsute female saints.^41^

Geiler von Kaysersberg’s Wild Man categories reflect a nuanced awareness of malnutrition-induced temporary hirsuteness absent from modern scholarship. Furthermore, I here suggest, a comparable subliminal understanding of connections between hirsuteness and starvation underpins the depiction of some anchorites, hermits or saints who spent long periods in malnourished religious contemplation as hairy, or the perception of medieval Europeans that some of the impoverished, malnourished men and women lurking on the plague-ridden, forested edges of their lands as hairy Wild Men. Recognition of the significance of this medical symptom illuminates the Wild Man and Wild Woman’s acquisition, during the famine-ridden twelfth century, of their defining hairiness, adding another layer of complexity to the early modern reception of pathologically hirsute women.

## 4. Barbara Urslerin

Unlike the Tuscan girl or the Gonzalez family, no noble protection was afforded to Barbara Urslerin, the only sufferer of hypertrichosis born in seventeenth-century Europe known to have survived into adulthood. Her Bavarian parents showed her around European fairgrounds for money from earliest infanthood, and she continued to tour widely with her husband-manager. Her activities are recorded in far more detail than any other pre-modern hypertrichosis case. One document previously overlooked in this context is *Ein erschröckliche / vnd doch warhafftige Newe Zeitung / von einer erschröcklichen Mißgeburt*, an illustrated single-leaf broadsheet by Christoph Kraus published in Kempten in 1629, recording an unidentified newborn. I identify this as a previously unknown record of Urslerin’s birth and birth date, published to be sung in public, and sold as a souvenir of their performance, by itinerant German fairground news-singers. Explicitly linking the depicted baby’s appearance to the Wild Man tradition, it documents it as ‘gar ein schröckliche Mißgeburt’, born to unnamed parents in the village of Mursellers near Kempten, Bavaria on 16 February 1629:

Gantz harig ist zu sehen an /

so gar von wilder arte /

ein grosses Köpfflein thut es han /

mit einem knöbel Barthe /

Ist doch ein Mägdlin zu der frist /

wie es hie abgebildet ist /

vnnd jederman kan anschawen. /

Es lebt auch noch zu dieser zeit /

darzu frisch und gesunde /

was vns das Kind bedeutten thut /

wirdt bringen zeit vnnd stunde.^42^

Perhaps the earliest scientific eyewitness commentary on Urslerin is a report of an unnamed, bearded, three year-old girl with all-over body hair. A published medical case study of the Jewish physician Zacutus Lusitanus (1575–1642), it suggests that by 1632 the three year-old girl was a lucrative public attraction, professionally managed by itinerant show-people:

*De Monstris. Puella barbata*. Pvellam vidi ætatis trium annorum, formosam, & pulchram cum barba magna, cui totum corpus nimis hirsutum erat, & ex eius auribus, pili crassi, hirti, numerosi prodibant, ita oblongi, vt palmum & dimidium æquarent. Hoc monstrum circulatores lucrandi causâ publicè ostentabant.^43^

Thomas Bartholin, who repeatedly examined Urslerin, reports that as a six-year-old child in Copenhagen and subsequently in Belgium, she was still being shown around Europe by her parents.^44^ Presumably, the family avoided Germany because of the Thirty Years War. By the time Bartholin’s medical student Georg Seger viewed her in 1655, ‘*Barbara* […] *Balthasaris Ursleri* filia’ was giving her age as twenty-two years. He notes that in addition to blond, soft, curly hair all over her face and body, she had a thick beard reaching down to her belt (much shorter in the accompanying illustration), and was married but childless,^45^ although some later sources record a non-hirsute son. In 1656, the French physician Peter Borel describes Urslerin’s beard as long and white, likening it to that of a venerable old man of eighty.^46^ At an Antwerp carnival fair of the 1650s, Margaret Cavendish saw a woman: ‘like a Shagg-dog, not in Shape, but Hair, as Grown all over her Body, which Sight […] troubled my Mind a Long time’.^47^ Learned physicians and scientists continued to flock to Urslerin’s public exhibitions, challenged and intrigued by the ways in which medical and lay perceptions of her hair blurred multiple boundaries. These were between young and old, the immature, mature and post-fertile, male and female, groomed and unkempt, law-abiding, civilised citizens and natural, free Wild Men, familiar and foreign, even, and most disturbingly, between the hunter and the hunted, the human, the bestial and the supernatural.

By 1628, John Earle rated ‘the Stories of some men of Tyburne, or a strange monster out of Germany’ the top attractions of itinerant British fairground news-singers, ‘chanted from market to market, to a vile tune, and a worse throat, whilst the poore Country wench melts like her butter to heare them’.^48^ German print culture offered seventeenth-century Europe’s richest source of depictions and descriptions of congenitally physically exceptional humans. Shaped no less by literary and other cultural influences than by the limits of their anthropological, pathological and other scientific knowledge, the records of hairy girls and bearded women considered here provide uneasy and unsettling counterpoints to stereotypical preconceptions of the feminine and its reception in early modern Germany.

## Footnotes

1 J.-P. Migne, *Sæculum XII. S. Hildegardis Abbatissæ opera omnia*, Paris 1855, ‘Scivias seu Visiones: Visio sexta’, columns 505–534 (509). Thus paraphrased by Hildegard Strickerschmidt: ‘Die […] Gruppe hat behaarte Körper und eine schmutzbefleckte Seele, weil sie unkeusch und schamlos sind’ (*Geerdete Spiritualität bei Hildegard von Bingen: neue Zugänge zu ihrer Heilkunde*, Berlin 2008, p. 222).

2 *Twelfth Night*, III.iv.

3 Edward Webbe, *The rare and most wonderfull things which Edw. Webbe an Englishman borne, hath seene and passed in his troublesome trauailes*, London 1590, sig. B4.

4 Matteo Villani, *Cronica di Matteo Villani*, Florence 1825, III, p. 77 (Libro Quinto, cap. LIII: ‘D’una fanciulla pilosa presentata all’imperadore’).

5 Kenneth A. R. Kennedy, *God-apes and Fossil Men: Paleoanthropology of South Asia*, Ann Arbor 2000, p. 76

6 Henry Yule (ed. and tr.), ‘Recollections of travel in the East, by John de’ Marignolli, Papal Legate to the court of the Great Khan and afterwards Bishop of Bisignano’, *Cathay and the Way Thither: Being a Collection of Medieval Notices of China*, 2 vols, London 1866, II, pp. 335–94 (pp. 379–83).

7 Michel de Montaigne (tr. John Florio), *Essayes*, London 1613, p. 45.

8 *.Jardín de flores curiosas*, Madrid 1570. This quotation: Antonio de Torquemada (tr. Lewis Lewkenor), *The Spanish Mandeuile of miracles*, London 1600, sig. 10.

9 Torquemada, *Miracles*, sig. 10.

10 Nathaniel Wanley, *The wonders of the little world*, London 1678, p. 7.

11 Jody Enders, ‘The Devil in the Flesh of Theater’, *Transformationen des Religiösen: Performativität und Textualität im geistlichen Spiel*, ed. Ingrid Kasten and Erika Fischer-Lichte, Berlin 2007, pp. 127–38 (pp. 130–32).

12 Eberhard Werner Happel, *Relationes curiosae. Oder Denckwürdigkeiten der Welt: […] daß also diese Arbeit gar füglich E. G. Happelii Continuation seiner[…] curieusen Relationen genannt werden könne*, Hamburg 1707, I, pp. 427–8, illustrated.

13 Jean Froissart, *Here Begynneth the Thirde and Fourthe Boke of Sir Iohn Froissart*, London 1525, sigs. ccxliii–ccxliiii: ‘Of the aduenture of a daunce that was made at Parys in lykenesse of wodehowses / wherin the Frenche Kynge was in parell of dethe’. Original illustration: Froissart, *Chroniques* IV, part 2, illuminated manuscript, Bruges *c*.1470. London, British Library, Harley 4380, f. 1r.

14 *Ein ser erschröckliche doch warhafftige Geschicht /so sich begeben hat in dem landt zü Wirdenberg /auff dem schloß Waldenberg genand*, broadsheet, Nuremberg 1570 (reproduced: Walter L. Strauss, *The German Single-Leaf Woodcut, 1550–1600: A Pictorial Catalogue. Vol. 3: S–Z*, New York 1975, p. 1337).

15 Martin Grundmann, *Neu-eröffnete Geist- und Weltliche Geschicht-Schule*, Görlitz 1677, pp. 191–3; Georg Wesenigk, *Das Spiel-süchtige / sieben-fächtige Polysigma der Bösen Spiel-Sieben*, Dresden 1702, pp. 81–3; Johann Georg Theodor Gräße, *Der Sagenschatz des Königreichs Sachsen*, Dresden 1855, pp. 58–9.

16 Ambroise Paré, *The workes of that famous chirugion Ambrose Parey translated out of Latine*, London 1634, p. 978.

17 Ulisse Aldrovandi, *Monstrorum historia. Cum Paralipomenis historiae omnium animalium*, Bologna 1642, p. 579 (Caput VII, Icone I: ‘Infans Aethiops, & virgo villosa’, woodcut).

18 M. A. Katritzky, ‘Images of ‘monsters’ and performers: J. A. Comenius’s *Orbis pictus* and *Aristotle’s masterpiece*’, in *Practicing New Editions: Transformation and Transfer of the Early Modern Book, 1450–1800*, ed. Hiram Kümper and Vladimir Simić, Nordhausen 2011, pp. 77–118.

19 Max Bartels, ‘Ueber abnorme Behaarung beim Menschen I *Zeitschrift für Ethnologie*, 8 (1876), 110–29; II, *ZfE*, 11 (1879), 145–94; III, *ZfE*, 13 (1881), 213–33 (see especially II, 169 and for the tables: I, 124 and II, 182).

20 Wanley, *Wonders*, p. 7.

21 Roberto Zapperi, *Der wilde Mann von Teneriffa: Die wundersame Geschichte des Pedro Gonzalez und seiner Kinder*, Munich 2004, pp. 46–7, 81–2, 115, 215.

22 Munich, Bayerisches Hauptstaatsarchiv, Geheimes Hausarchiv, Fürstensachen 426/I: 10 March 1571; Berndt Ph. Baader, *Der bayerische Renaissancehof Herzog Wilhelms V. (1568–1579)*, Leipzig 1943, p. 74.

23 Munich, Bayerisches Hauptstaatsarchiv, Geheimes Hausarchiv, Korr.Akt 606 V, f.212v (file draft of letter sent). See also Zapperi, *Der wilde Mann*, pp. 194–5; Merry E. Wiesner-Hanks, *The Marvelous Hairy Girls: The Gonzales Sisters and Their Worlds*, New Haven 2009; M. A. Katritzky, *Healing, Performance and Ceremony in the Writings of Three Early Modern Physicians, Hippolytus Guarinonius and the brothers Felix and Thomas Platter*, Farnham 2012, pp. 206–7.

24 Eberhard Werner Happel, *E[verhardus]G[uernerus] Happelii Gröste Denckwürdigkeiten der Welt: oder so genannte Relationes curiosae*, 5 vols, Hamburg 1683, 1685, 1687, 1689, 1691, II: engraved plate opposite p. 312.

25 Happel, *Relationes curiosae*, II, 1685, p. 316 (the first of two, as pp. 313–16 are erroneously repeated).

26 Thomas D’Urfey, *Collins Walk through London and Westminster. A Poem in Burlesque*, London 1690, pp. 201–2.

27 Aldrovandi, *Monstrorum historia*, pp. 16–18. On the Gonzalez family, see also pp. 473, 580.

28 Richard Bernheimer, *Wild Men in the Middle Ages: A Study in Art, Sentiment, and Demonology*, Cambridge 1952, p. 1.

29 Timothy Husband, *The Wild Man: Medieval Myth and Symbolism*, New York 1980, p. 1.

30 Johann Geiler von Kaysersberg, *Die Emeis*, Strassburg 1517, sigs. XXXIXv–XXXXIr: ‘solitari’, ‘sachanni’, ‘pigineni’, ‘diaboli’, ‘Hyspani’.

31 See, for example, Francesca Hornak, ‘History of the world in 100 modern objects: Yoga mat’, *Sunday Times* (‘Style’, 30 June 2013), 18: ‘Since her reawakening, Sophie […] is nut-brown and 2st lighter than before, with an outie bellybutton, lotus tattoos and downy hair on her face.’

32 Renata Strumia, ‘Skin signs in anorexia nervosa’, *Dermato-Endocrinology*, 1/5 (2009), 268–70.

33 See, for example, his woodcarving of *c*. 1490 (Bayerisches Nationalmuseum, illustrated: Roberta Milliken, *Ambiguous Locks: An Iconology of Hair in Medieval Art and Literature*, Jefferson, NC 2012, p. 196).

34 Discussed (without reference to anorexia) by Brett D. Hirsch, ‘“What are these faces?” Interpreting bearded women in *Macbeth*’, in *Renaissance Poetry and Drama in Context*, ed. Andrew Lynch and Anne M. Scott, Newcastle-upon-Tyne 2008, pp. 91–113 (97); and Helen King, *The One-Sex Body On Trial: The Classical and Early Modern Evidence*, Farnham 2013, pp. 78–9.

35 *Das Heldenbuch mit synen figuren*, Strasbourg 1509, sig. k.ii.

36 Ilse E. Friesen, *The Female Crucifix: Images of St Wilgefortis since the Middle Ages*, Waterloo 2001, p. 119.

37 I. E. Bush and V. B. Mahesh, ‘Adrenocortical hyperfunction with sudden onset of hirsutism’, *Journal of Endocrinology*, 18 (1959), 1–25; Harry S. Lipscomb and Hebbel E. Hoff, ‘Saint Uncumber or La Vierge Barbue’, *Bulletin of the History of Medicine*, 37 (1963), 523–7 (526).

38 Janet T. Schulenberg, *Forgetful of their Sex: Female Sanctity and Society, ca. 500–1100*, Chicago 1998, pp. 152–3.

39 J. Hubert Lacey, ‘Anorexia nervosa and a bearded female saint’, British Medical Journal, 285 (1982), 1816–17.

40 Rudolph M. Bell, *Holy Anorexia*, Chicago 1985.

41 Hirsch, ‘Bearded women’, p. 96.

42 Nuremberg, Germanisches Nationalmuseum. Illustrated: Eugen Holländer, *Wunder, Wundergeburt und Wundergestalt in Einblattdrucken des fünfzehnten bis achtzehnten Jahrhunderts; kulturhistorische Studie*, Stuttgart 1921, p. 277; Dorothy Alexander, and Walter L. Strauss, *The German Single-Leaf Woodcut, 1600–1700: A Pictorial Catalogue*, vol. 1: A–N, New York 1977, p. 338.

43 Zacutus Lusitanus, *Praxis medica admiranda*, Lyon 1637, p. 504.

44 Thomas Bartholin, *Historiarum Anatomicarum Rariorum, Centuria I et II*, Amsterdam 1654, p. 62 (‘Historia XLII: Puella hirsuta & barbata’).

45 Georg Seger, ‘Observatio XVC: De muliere hirsuta et barbata’, *Miscellanea curiosa sive ephemeridum medico-physicarum Germanicarum Academiæ*, Nuremberg 1693, p. 246.

46 Peter Borel, *Historiarum et Observationum Medicophysicarum, centuriæ IV*, Paris 1656, p. 15.

47 Margaret Cavendish, *CCXI sociable letters*, London 1664, p. 405; M. A. Katritzky, *Women, Medicine and Theatre 1500–1750: Literary Mountebanks and Performing Quacks*, Aldershot 2007, pp. 2–4.

48 John Earle, *Micro-cosmographie or, a peece of the world discovered*, London 1628, sig. E10.

